# Supplementary material for: Intraspecies associations from strain-rich metagenome samples
Source: bioRxiv. 2025 Feb 8:2025.02.07.636498. Preprint. [Version 1] doi: 10.1101/2025.02.07.636498 (PMC11839054; doi:10.1101/2025.02.07.636498)
Supplement: Supplement 4 [file NIHPP2025.02.07.636498v1-supplement-4.pdf]

bioRxiv preprint doi: https://doi.org/10.1101/2025.02.07.636498; this version posted February 8, 2025. The copyright holder for this preprint (which was not certified by peer review) is the author/funder, who has granted bioRxiv a license to display the preprint in perpetuity. It is made available under aCC-BY 4.0 International license.

Supplemental Figures

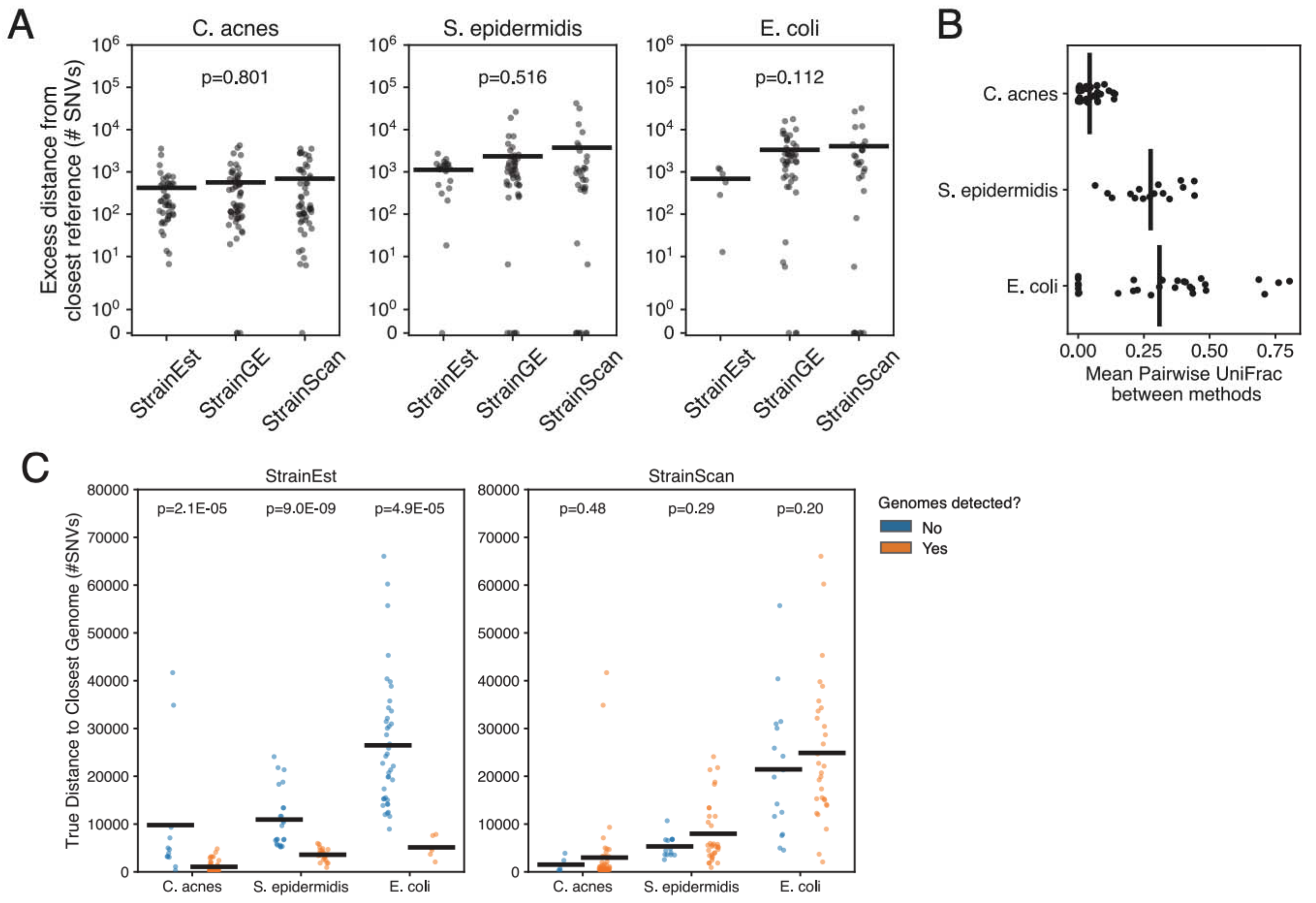

Figure S1: Behavior of strain-level classification algorithms when reference databases are systematically missing diversity. Results of three existing strain-level profiling methods when a reference database missing a section of phylogenetic diversity is used to classify a random held-out genome subsampled to 10X coverage across the reference genome. In the majority of simulations, the held-out genome is detected as one or more known genomes. (A) Excess Distance (Methods) between the true held-out genome and the detected genome(s), compared to the distance between the held-out genome and its true closest reference in the database. P-value shown is the result of a Kruskal-Wallis test for difference between methods. (B) Consistency in method outputs, as measured by the mean pairwise UniFrac distance between outputs of each method. For *E. coli*, this metric was only calculated between StrainGE and StrainScan, as StrainEst did not return an output for a majority of simulations. (C) More novel strains, as measured by the distance (# SNVs) to the closest reference genome, are less likely to be detected as a known genome by StrainEst, but not StrainScan or StrainGE (no graph shown for StrainGE as genomes were detected in all simulations).

bioRxiv preprint doi: https://doi.org/10.1101/2025.02.07.636498; this version posted February 8, 2025. The copyright holder for this preprint (which was not certified by peer review) is the author/funder, who has granted bioRxiv a license to display the preprint in perpetuity. It is made available under aCC-BY 4.0 International license.

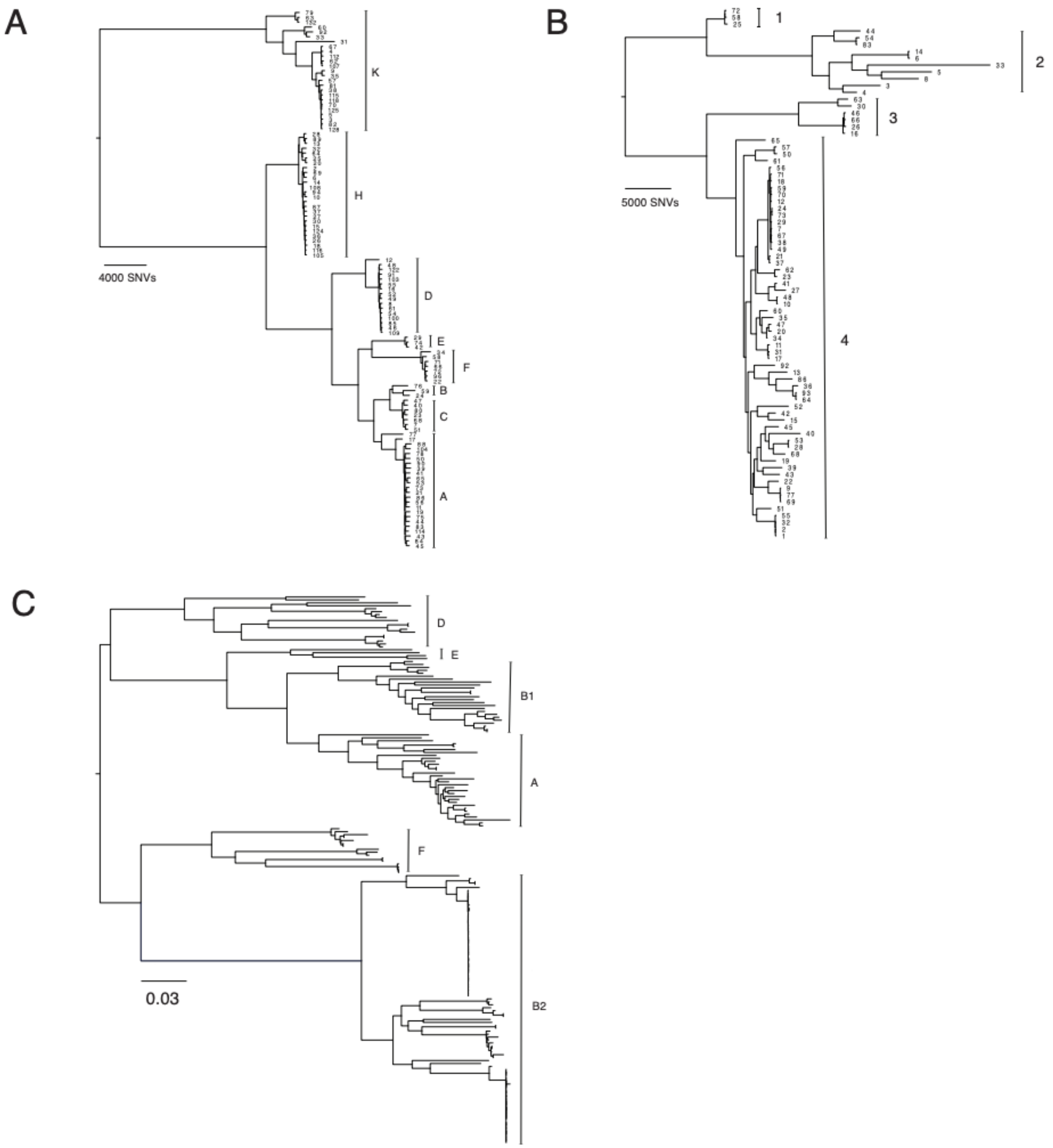

Figure S2: Phylogenies for *C. acnes*, *S. epidermidis, E. coli* used in held-out clade simulations. Core-genome maximum likelihood phylogenies (constructed using RaXML v.8.2.12) for (A) *C. acnes*, (B) *S. epidermidis,* and (C) *E. coli*. Lineages are labelled with a number, while phylogroups represent major intraspecies clades and are labelled with bars. Representative genomes were chosen for each lineage based on highest sequencing depth.

bioRxiv preprint doi: https://doi.org/10.1101/2025.02.07.636498; this version posted February 8, 2025. The copyright holder for this preprint (which was not certified by peer review) is the author/funder, who has granted bioRxiv a license to display the preprint in perpetuity. It is made available under aCC-BY 4.0 International license.

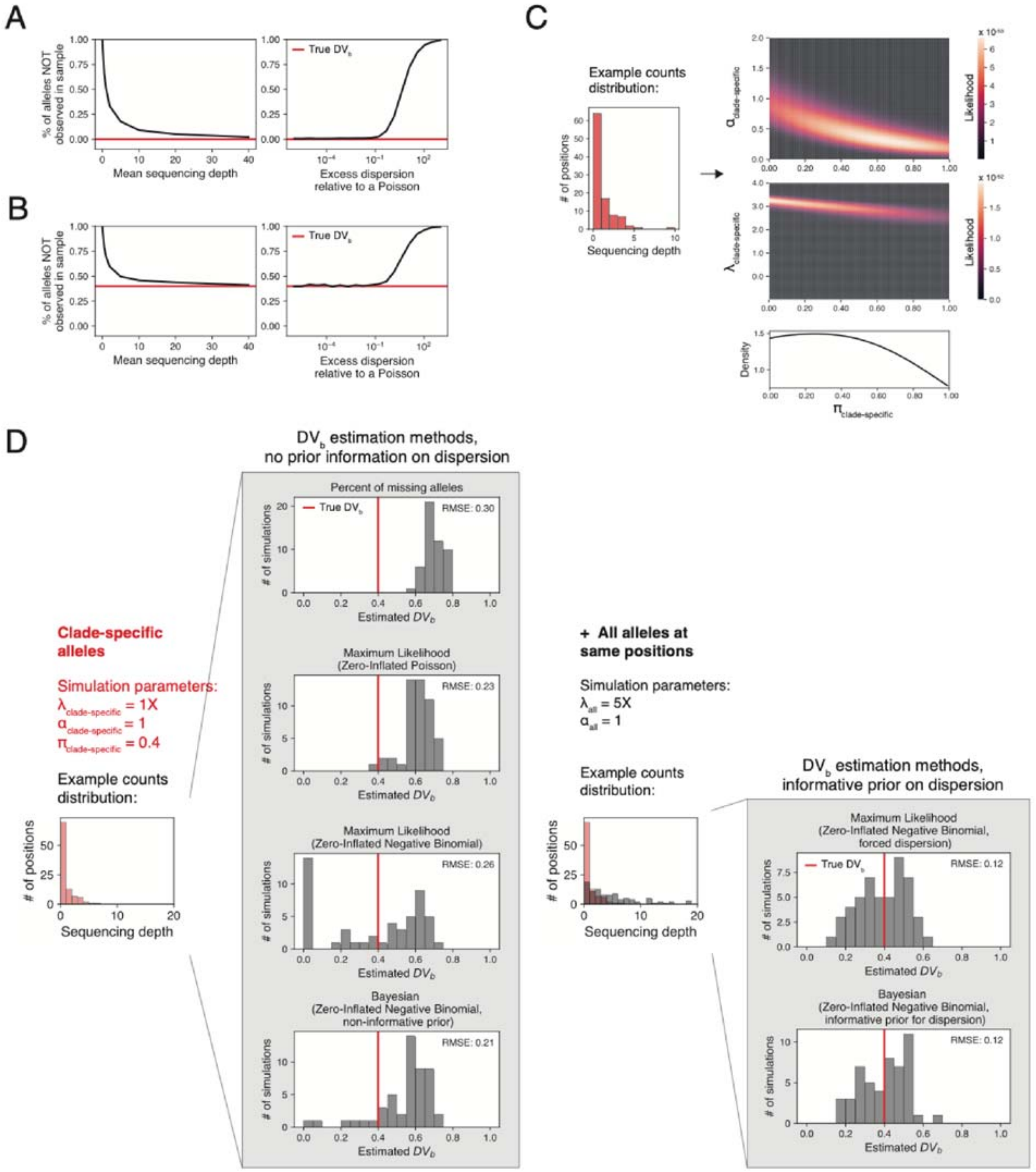

Figure S3: Prior information on read dispersion helps to constrain DV_b estimates. (A-B) The portion of missing clade-specific alleles does not necessarily reflect true values of DV_b. We generated hypothetical read counts across a set of clade-specific alleles by simulating from a zero-inflated negative binomial (ZINB) distribution, varying either sequencing depth (left; from 0.1X to 40X) or overdispersion relative to a Poisson (right; from 10^{-6} to 10^{3}). Values shown are the mean of 50 simulations per parameter set. The observed portion of missing clade-specific alleles begins to diverge from the true DV_b at sequencing depth <10X and overdispersion relative to a Poisson > 0.5. (C) When

bioRxiv preprint doi: https://doi.org/10.1101/2025.02.07.636498; this version posted February 8, 2025. The copyright holder for this preprint (which was not certified by peer review) is the author/funder, who has granted bioRxiv a license to display the preprint in perpetuity. It is made available under aCC-BY 4.0 International license.

just using information on the number of clade-specific markers, a wide range of possible parameter combinations can reasonably explain any given counts distribution. Left: A random distribution of read counts supporting a set of clade-specific alleles. Right: Heatmap of the ZINB likelihood given the left distribution along parameters $\alpha_{clade\text{-}specific}$, $\pi_{clade\text{-}specific}$, and $\lambda_{clade\text{-}specific}$ (see Fig. 2B). The marginal density of $\pi_{clade\text{-}specific}$ (bottom right) supports a wide range of plausible values. (D) PHLAME overcomes this uncertainty by setting a prior on dispersion (represented by the parameter $\alpha_{clade\text{-}specific}$) using the coverage of all alleles at the same positions. Left: Attempts to measure the true DV_b value using without prior knowledge of the dispersion returns inaccurate results at low depth and realistic overdispersion. We simulated 50 hypothetical read count distributions from a ZINB distribution, then measured DV_b using the various methods, including the proportion of missing alleles, a maximum-likelihood zero-inflated Poisson and ZINB model, as well as a Bayesian ZINB model without an informative prior. The root mean squared error of each method is shown next to each plot. Right: Including prior information on the dispersion improves accuracy in the same simulation set. Here, we additionally simulated a read count distribution to represent the coverage of all alleles at the same set of positions as the clade-specific alleles. Including this information in either a maximum-likelihood or Bayesian inference algorithm improves RMSE compared to estimating DV_b from only the counts distribution across clade-specific alleles.

bioRxiv preprint doi: https://doi.org/10.1101/2025.02.07.636498; this version posted February 8, 2025. The copyright holder for this preprint (which was not certified by peer review) is the author/funder, who has granted bioRxiv a license to display the preprint in perpetuity. It is made available under aCC-BY 4.0 International license.

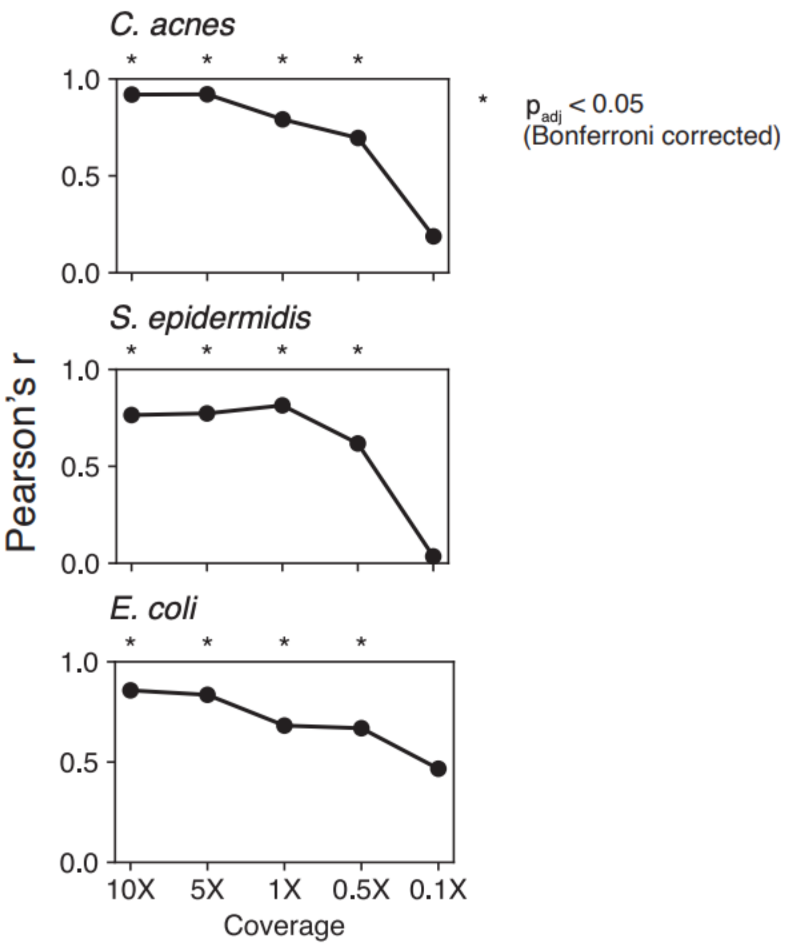

Figure S4: Accuracy of metagenomic estimates of DV_b with varying per-clade coverage. Pearson's correlation coefficient between metagenomic point estimates of DV_b (represented by the parameter π) and ground truth DV_b values determined from the species phylogeny. Metagenomic inferences were obtained by constructing a reference database in which a single clade and all its descendants were held out, then using that database to classify a simulated metagenome containing a single genome form the held-out clade (See Fig. 2E). Simulated metagenomes were run through default PHLAME classification parameters, which requires minimum of 10 positions to have at least one read supporting the clade-specific allele. Low-coverage simulations that did not fulfill this criterion were not included in correlation calculations. While inference accuracy decreased at lower coverage, significant correlations (shown with stars) were recovered at per-clade coverages as low as 0.5X.

bioRxiv preprint doi: https://doi.org/10.1101/2025.02.07.636498; this version posted February 8, 2025. The copyright holder for this preprint (which was not certified by peer review) is the author/funder, who has granted bioRxiv a license to display the preprint in perpetuity. It is made available under aCC-BY 4.0 International license.

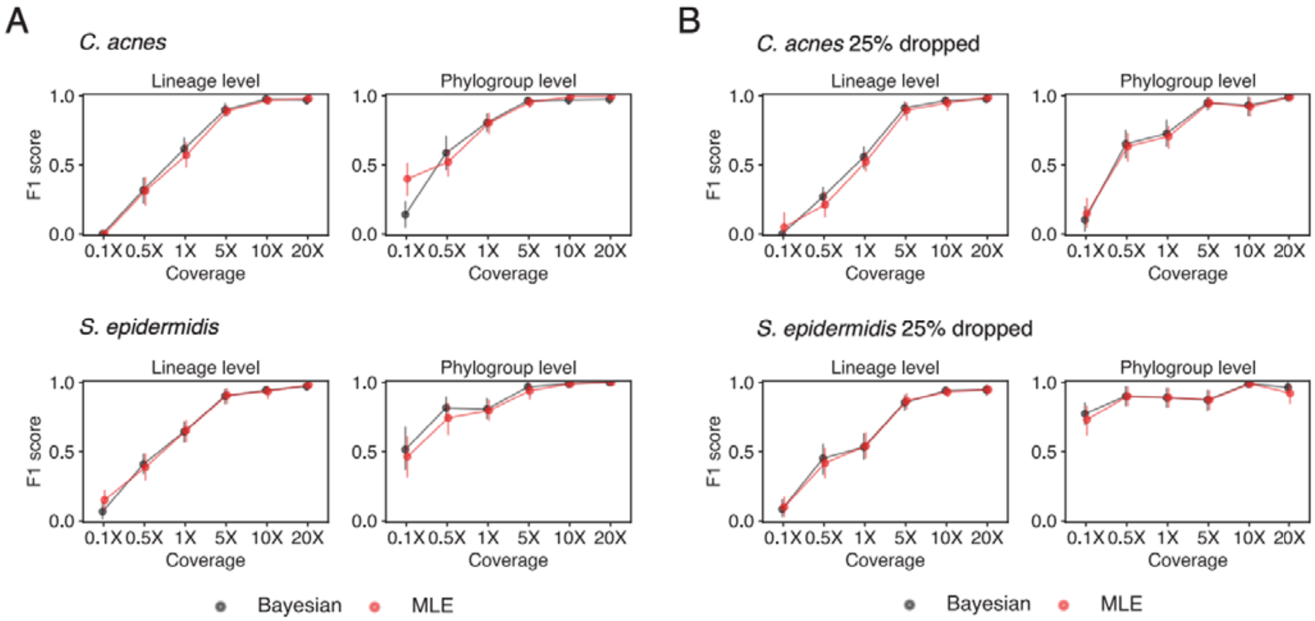

Figure S5: Minimal decrease in performance between Bayesian and Maximum Likelihood implementations of PHLAME model. We compared performance between Bayesian and maximum likelihood implementations of the PHLAME model (Supplemental Methods) using simulated metagenome benchmarks (see Fig. 3). In the Bayesian implementation, we estimated full posterior distributions over π and required 50% of the posterior distribution to be below 0.35 in order for a clade to count as detected. In the maximum likelihood implementation, we only inferred a point estimate on π and required this estimate to be below 0.35 in order for a clade to count as detected. Across species and simulations, results between the Bayesian and maximum likelihood implementations are largely consistent, with the maximum likelihood approach achieving only a minimal decrease in F1 score.

bioRxiv preprint doi: https://doi.org/10.1101/2025.02.07.636498; this version posted February 8, 2025. The copyright holder for this preprint (which was not certified by peer review) is the author/funder, who has granted bioRxiv a license to display the preprint in perpetuity. It is made available under aCC-BY 4.0 International license.

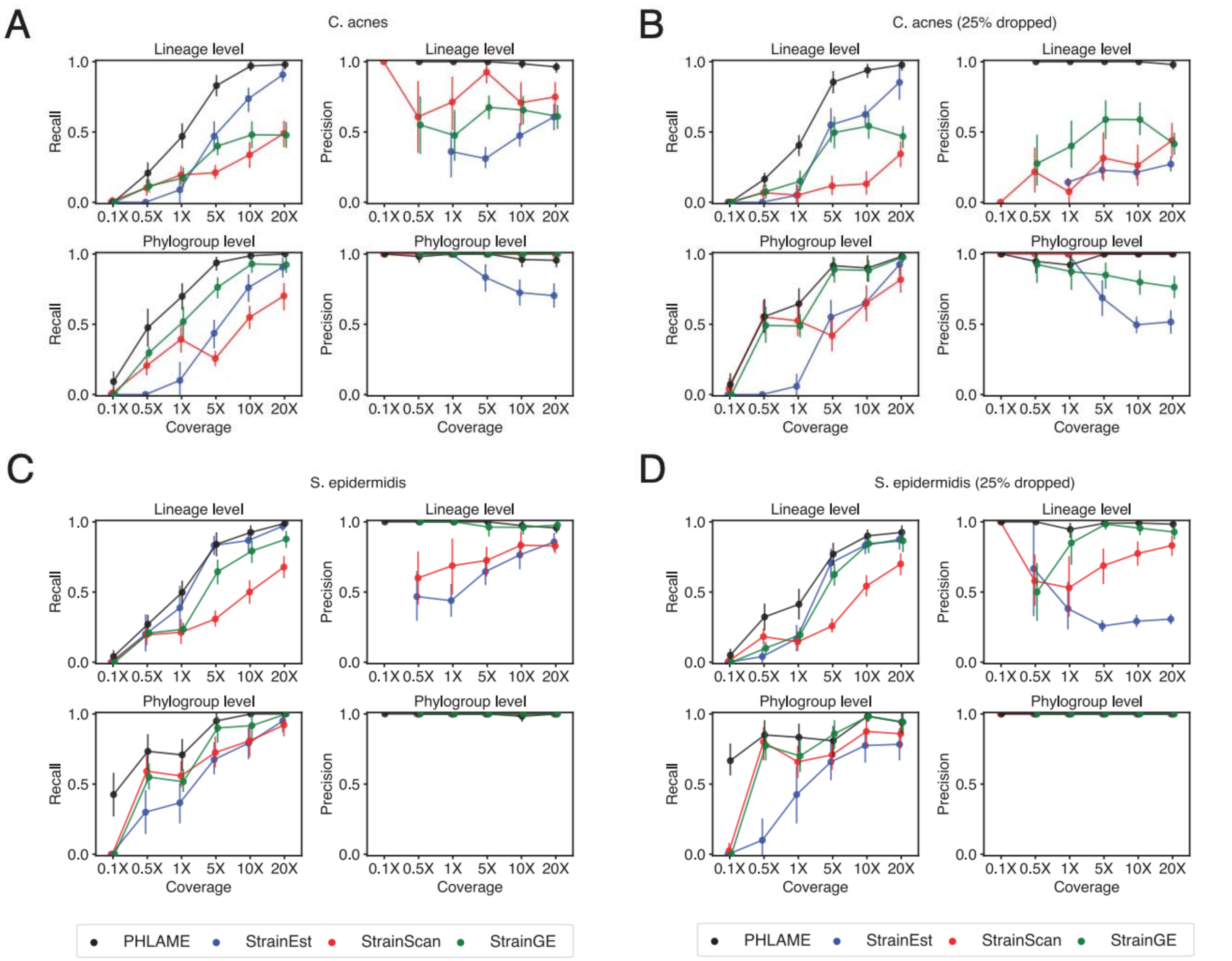

Figure S6: Precision and recall plots for synthetic metagenome benchmarks. Recall (left columns) and precision (right columns) for PHLAME, StrainEst, StrainGE, and StrainScan in synthetic metagenome benchmarks. Similar plots are shown for databases for (A) *C. acnes* with a perfect database; (B) *S. epidermidis* with a perfect database; (C) *C. acnes* with 25% of the clades held out; and (D) *S. epidermidis* with 25% of the clades held out. F1 scores are shown in Fig. 3. See also Fig. S7 and Fig. S8.

bioRxiv preprint doi: https://doi.org/10.1101/2025.02.07.636498; this version posted February 8, 2025. The copyright holder for this preprint (which was not certified by peer review) is the author/funder, who has granted bioRxiv a license to display the preprint in perpetuity. It is made available under aCC-BY 4.0 International license.

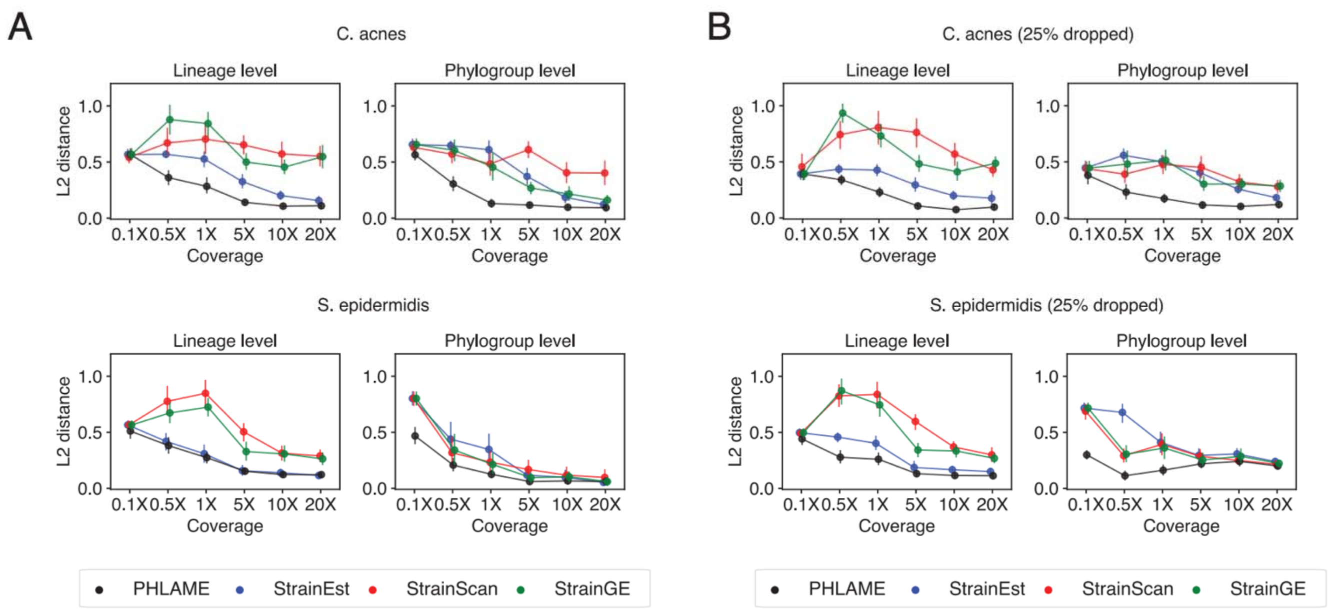

Figure S7: Relative abundance benchmarking using L2 distance. We measure accuracy in relative abundance estimates using L2 distance, which measures the Euclidean distance between the ground truth relative abundance vector and a given estimated relative abundance vector (lower is better). (A) L2 distance calculated for different methods in simulated metagenome benchmarks with a perfect *C. acnes* or *S. epidermidis* reference database. (B) L2 distance calculated for different methods in simulated metagenome benchmarks where 25% of the phylogroups are randomly held out.

bioRxiv preprint doi: https://doi.org/10.1101/2025.02.07.636498; this version posted February 8, 2025. The copyright holder for this preprint (which was not certified by peer review) is the author/funder, who has granted bioRxiv a license to display the preprint in perpetuity. It is made available under aCC-BY 4.0 International license.

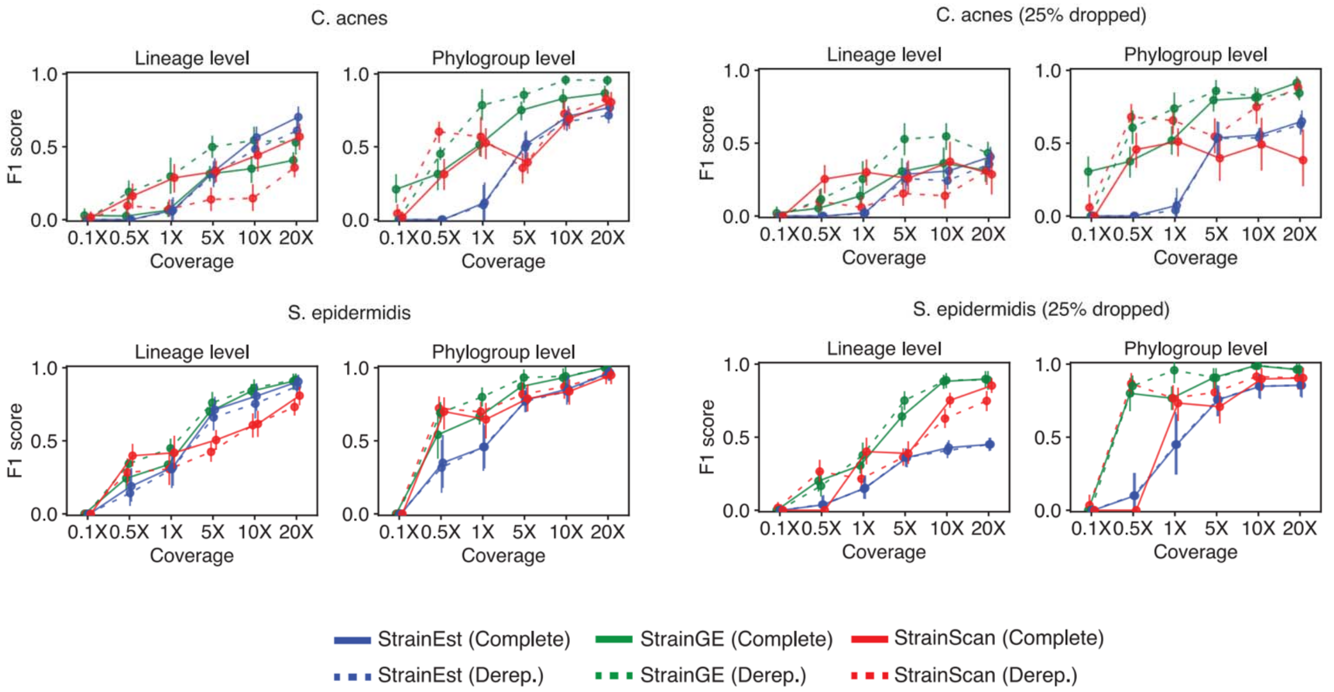

Figure S8: Performance between complete and dereplicated databases for StrainEst, StrainGE, and StrainScan. F1 score for each StrainEst, StrainGE, and StrainScan given either a complete database containing all reference genomes for a species (solid lines) or a dereplicated database containing a single representative genome per lineage (dashed lines).

bioRxiv preprint doi: https://doi.org/10.1101/2025.02.07.636498; this version posted February 8, 2025. The copyright holder for this preprint (which was not certified by peer review) is the author/funder, who has granted bioRxiv a license to display the preprint in perpetuity. It is made available under aCC-BY 4.0 International license.

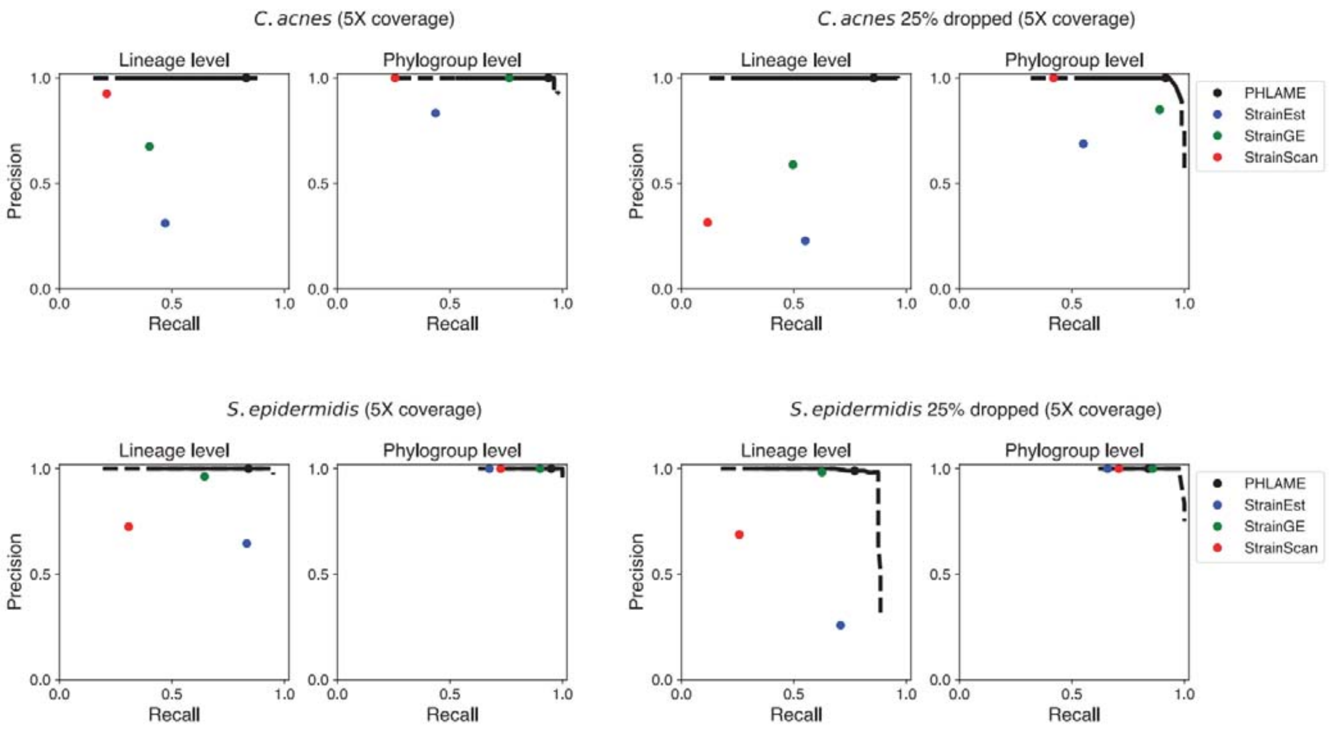

Fig S9: PHLAME results are robust to varying parameters. Precision-recall curves created by varying the detection threshold for PHLAME. The main detection threshold used for PHLAME requires at least 50% of the posterior density for π to be below a certain DV_b value. While possible values of DV_b range from 0 to 1, not all parameters are reasonable (for example, accepting detections when 50% of the posterior density for π is below 0.90 DV_b may be overly permissive). Focusing on the set of benchmarks where the focal species was subsampled to 5X coverage, we show ROC curves for PHLAME across the full range of possible π thresholds (0-1, dashed lines), as well as a set of possible π thresholds that we consider reasonable π thresholds in applied use (0.05-0.5, solid line). Mean precision and recall of StrainEst, StrainGE, and StrainScan for the same set of simulations are shown in colored dots, and mean precision and recall for the default PHLAME parameters shown in Figs. 3, S6-S7 is shown as a black dot.

bioRxiv preprint doi: https://doi.org/10.1101/2025.02.07.636498; this version posted February 8, 2025. The copyright holder for this preprint (which was not certified by peer review) is the author/funder, who has granted bioRxiv a license to display the preprint in perpetuity. It is made available under aCC-BY 4.0 International license.

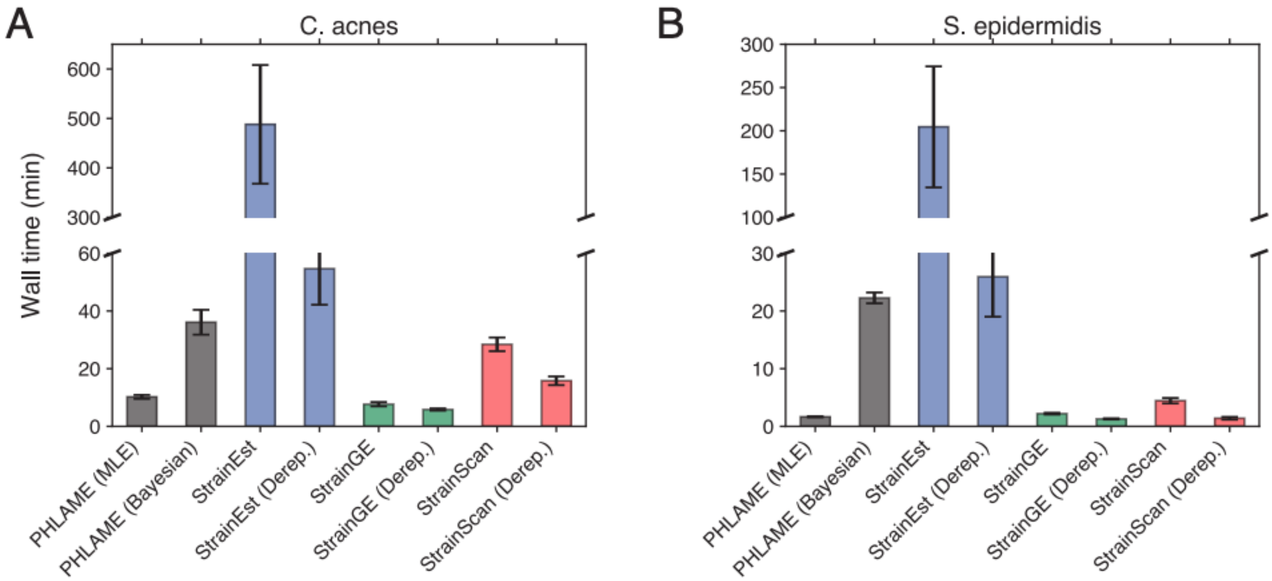

Figure S10: Comparison of method runtimes. Wall time (mean and 95% CI) to run PHLAME, StrainEst, StrainGE, and StrainScan. Read-to-result wall times were measured for (A) C. acnes and (B) S. epidermidis on the set of simulations where the focal species was at 20X coverage, and no genomes were held out from databases. For PHLAME and StrainEst, the time reported includes the time associated with read alignment and bam conversion. Each method was run on an AMD EPYC 7513 2.6GHz 64-core processor with 24Gb of allocated memory. Wall times were measured using the snakemake (v.7.20.0) benchmark utility.

bioRxiv preprint doi: https://doi.org/10.1101/2025.02.07.636498; this version posted February 8, 2025. The copyright holder for this preprint (which was not certified by peer review) is the author/funder, who has granted bioRxiv a license to display the preprint in perpetuity. It is made available under aCC-BY 4.0 International license.

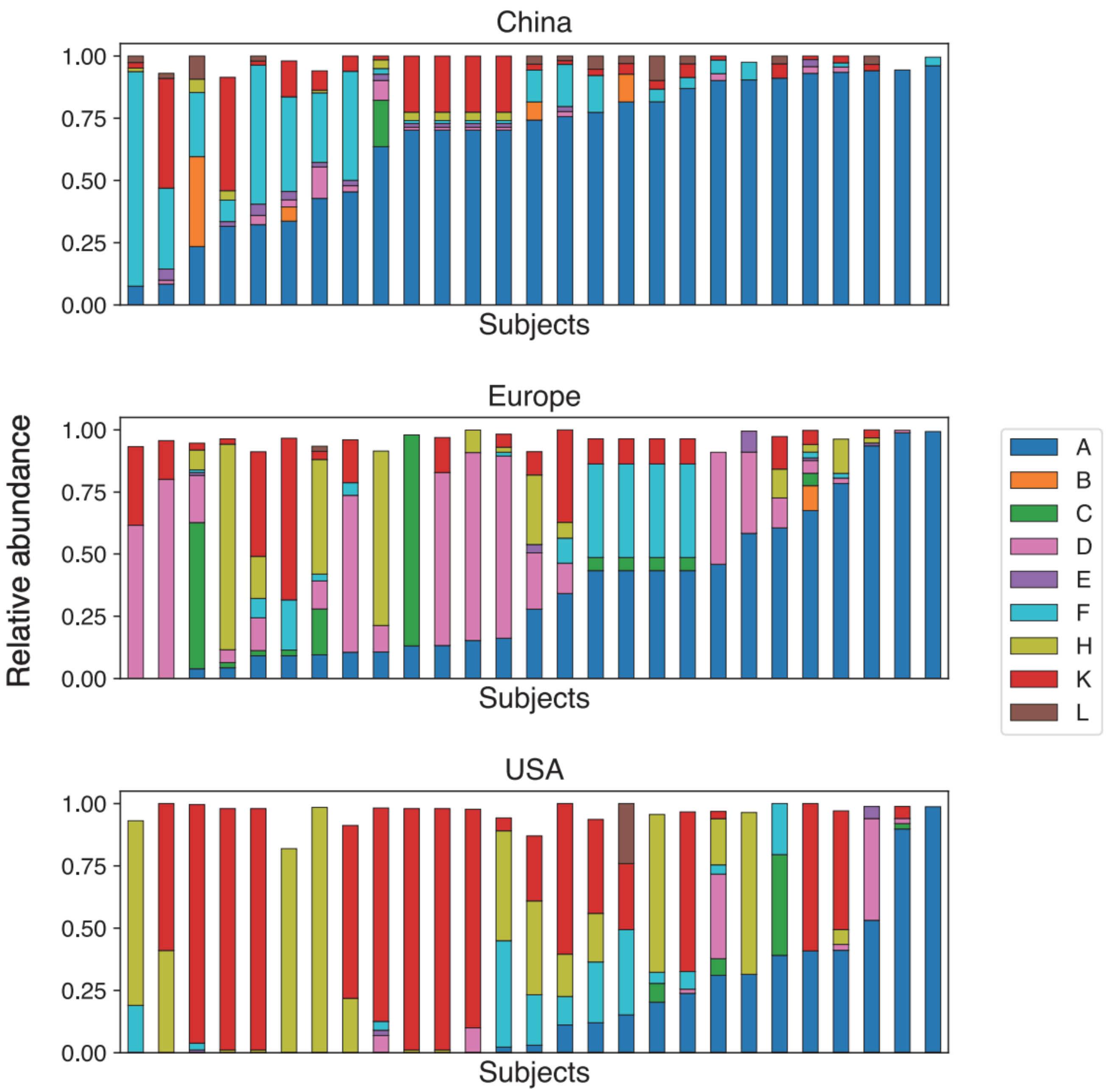

Figure S11: On-person taxonomic abundances of *C. acnes* phylogroups. 25 random taxonomic barplots per geographic region showing on-person *C. acnes* phylogroup abundances (one bar represents one subject).

bioRxiv preprint doi: https://doi.org/10.1101/2025.02.07.636498; this version posted February 8, 2025. The copyright holder for this preprint (which was not certified by peer review) is the author/funder, who has granted bioRxiv a license to display the preprint in perpetuity. It is made available under aCC-BY 4.0 International license.

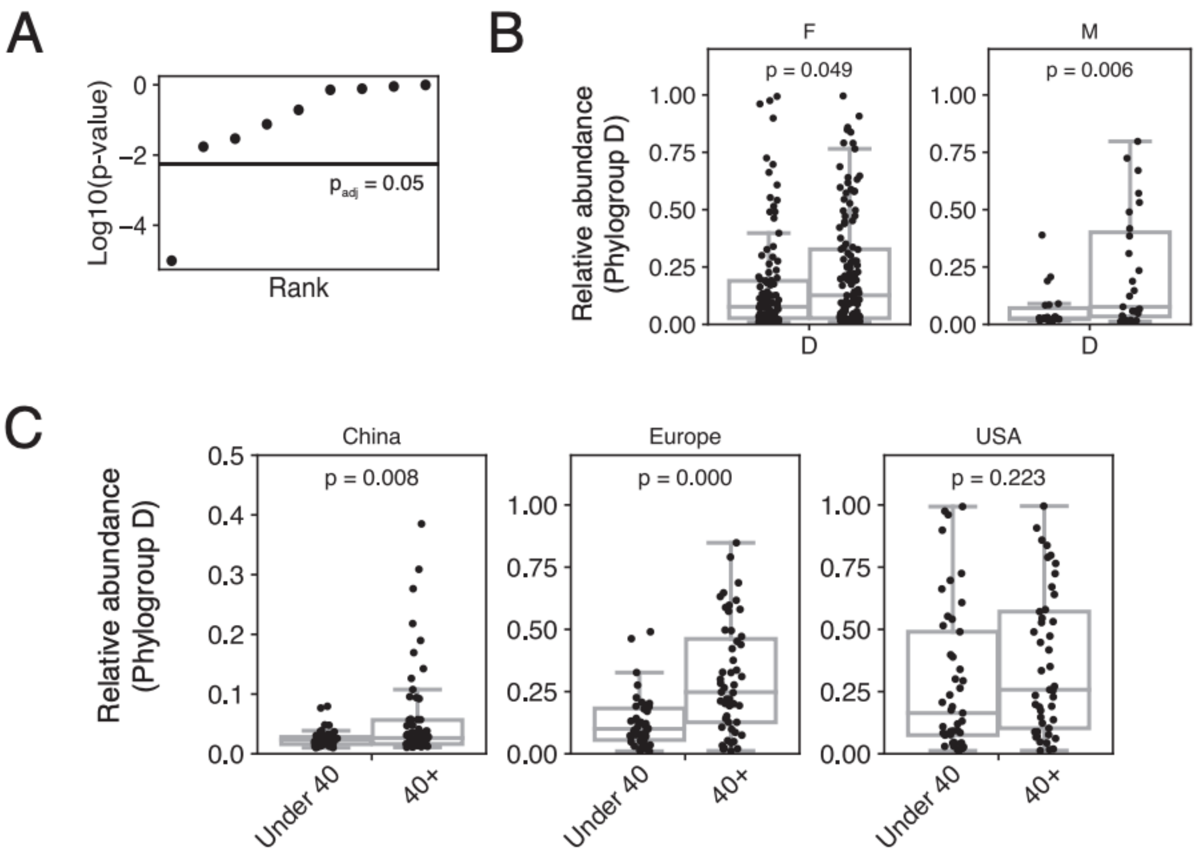

Figure S12: The relationship between *C. acnes* phylogroup D and age is robust across testable confounders. Multiple hypothesis correction for a rank-sum test for difference in phylogroup relative abundance between individuals under 40 compared to 40+. Black line represents an alpha of 0.05 after Bonferroni correction; only one phylogroup (phylogroup D) is significant after correction. (B) Difference in phylogroup D frequency on individuals under 40 compared to 40+, partitioned by reported sex. P-values represent the result of a Wilcoxon rank sum test. (C) Difference in phylogroup D frequency on individuals under 40 compared to 40+, partitioned by geographic region. P-values represent the result of a rank sum test. For the USA, this difference is not significant between individuals under 40 compared to 40+, but there is a significant rank correlation between phylogroup D and age (Fig. 5E).

bioRxiv preprint doi: https://doi.org/10.1101/2025.02.07.636498; this version posted February 8, 2025. The copyright holder for this preprint (which was not certified by peer review) is the author/funder, who has granted bioRxiv a license to display the preprint in perpetuity. It is made available under aCC-BY 4.0 International license.

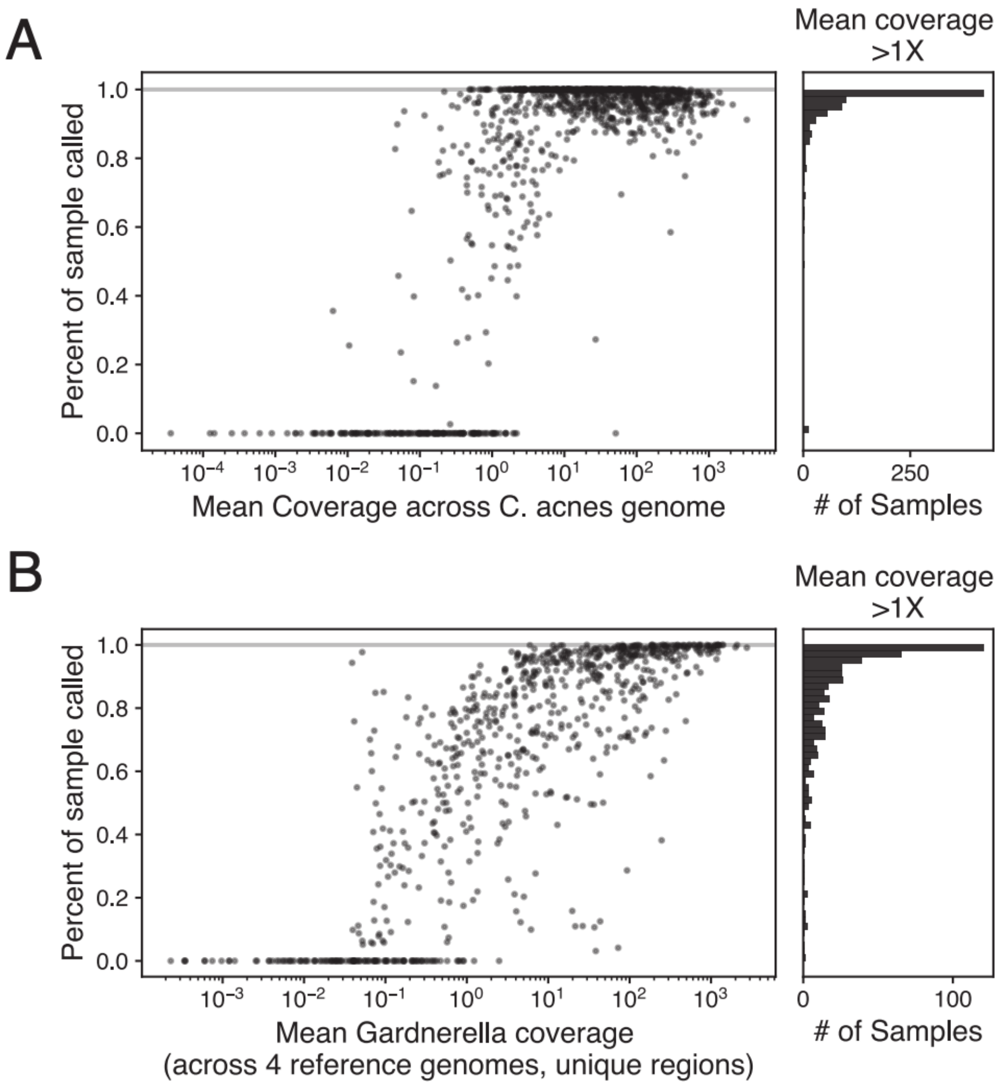

Figure S13: Novel *C. acnes* diversity is uncommon at the phylogroup level; novel *Gardnerella* diversity is more common. (A) Left: The percent of each sample classified by PHLAME at the phylogroup level as a function of mean coverage across the *C. acnes* genome (each dot represents one sample). Right: Histogram of the percent of sample classified by PHLAME at the phylogroup level, for only samples that reached greater than 1X mean coverage across the *C. acnes* genome. (B) Same diagram as (A) for *Gardnerella*. The coverage reported here is the mean coverage for each reference genome's unique regions, summed across the four reference genomes used to classify *Gardnerella* diversity. A larger proportion of the Gardnerella diversity is unclassifiable in vaginal microbiome samples with greater than 1X coverage, compared to *C. acnes* diversity in skin microbiome samples ($p < 0.001$ two sample K-S test).

bioRxiv preprint doi: https://doi.org/10.1101/2025.02.07.636498; this version posted February 8, 2025. The copyright holder for this preprint (which was not certified by peer review) is the author/funder, who has granted bioRxiv a license to display the preprint in perpetuity. It is made available under aCC-BY 4.0 International license.

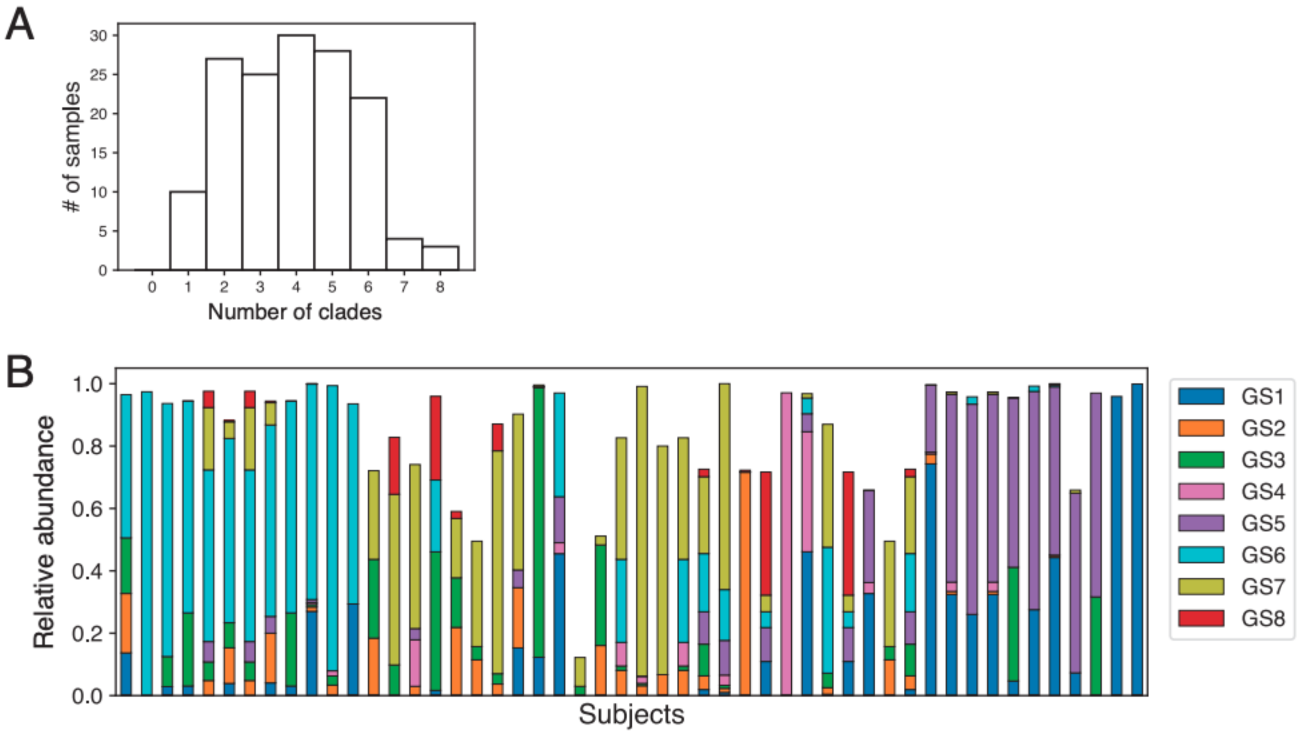

Figure S14: *Gardnerella* variation across subjects in the vaginal microbiome. (A) Number of *Gardnerella* clades (out of 8) per sample, detected across 149 subjects with > 3X coverage across 4 *Gardnerella* reference genomes (1 sample per subject). (B) Relative abundances of *Gardnerella* taxa in 50 random subjects with >3X coverage across 4 *Gardnerella* reference genomes (1 sample per subject).

bioRxiv preprint doi: https://doi.org/10.1101/2025.02.07.636498; this version posted February 8, 2025. The copyright holder for this preprint (which was not certified by peer review) is the author/funder, who has granted bioRxiv a license to display the preprint in perpetuity. It is made available under aCC-BY 4.0 International license.

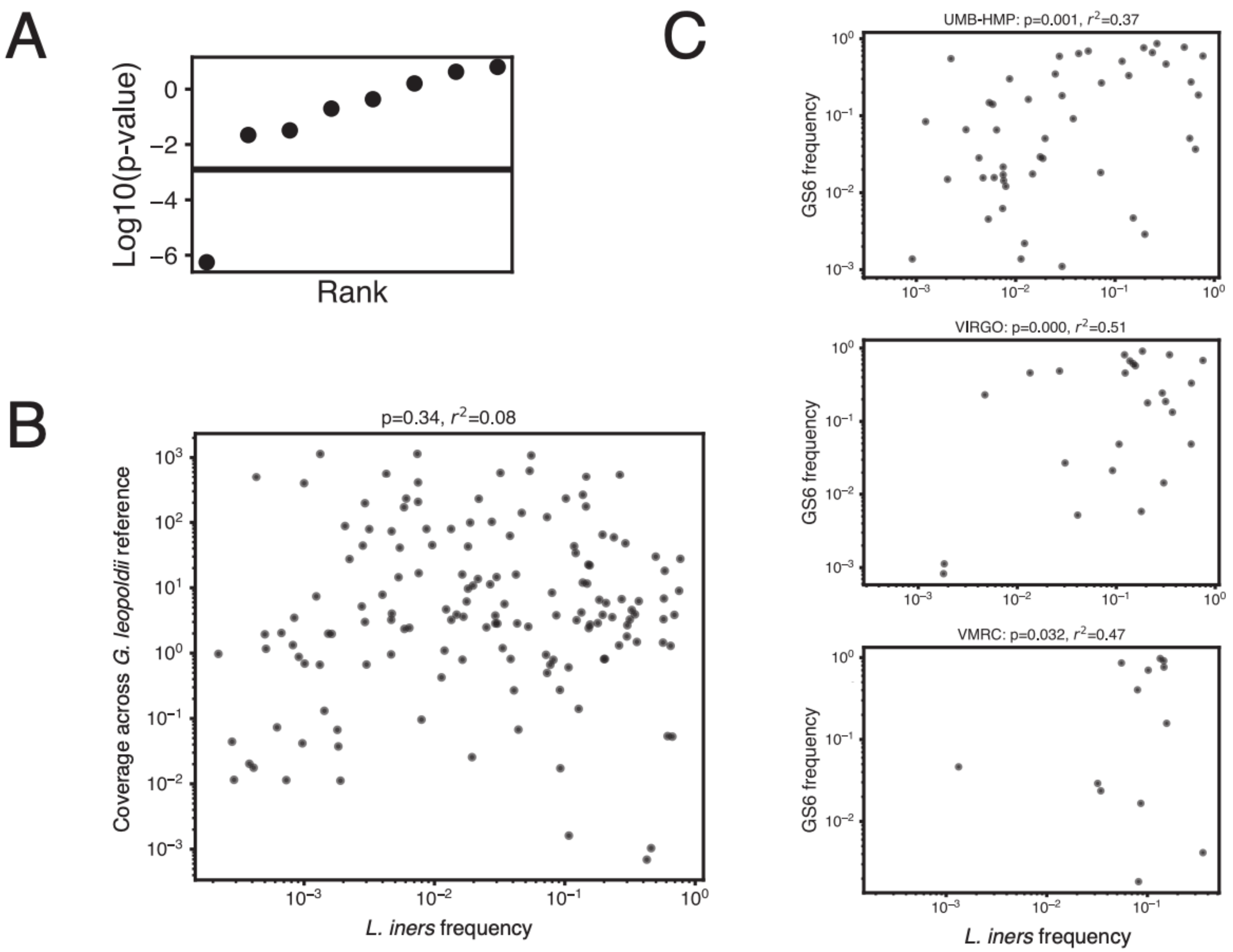

Figure S15: Association between GS6 (*G. swidsinskii*) and *L. iners* in the vaginal microbiome is consistent across possible confounders. (A) Multiple hypothesis correction (Spearman correlation) between *Gardnerella* clade frequency within the *Gardnerella* population and *L. iners* frequency in the sample. Black line represents a Bonferroni-corrected alpha of 0.01. After multiple hypothesis correction, only 1 clade (GS6) has a significant relationship with *L. iners* frequency. (B) There is no relationship between the frequency of *L. iners* in a sample and the number of reads mapping to the corresponding reference genome for GS6 (*G. leopoldii* 6420B), indicating that this association is not confounded by sequencing depth. Spearman's correlation coefficient and significance value are shown above the plot. (C) Association between the relative frequency of GS6 in the *Gardnerella* population and *L. iners* frequency in the species-level community remains significant across different studies (UMB-HMP, VIRGO, VMRC).

bioRxiv preprint doi: https://doi.org/10.1101/2025.02.07.636498; this version posted February 8, 2025. The copyright holder for this preprint (which was not certified by peer review) is the author/funder, who has granted bioRxiv a license to display the preprint in perpetuity. It is made available under aCC-BY 4.0 International license.

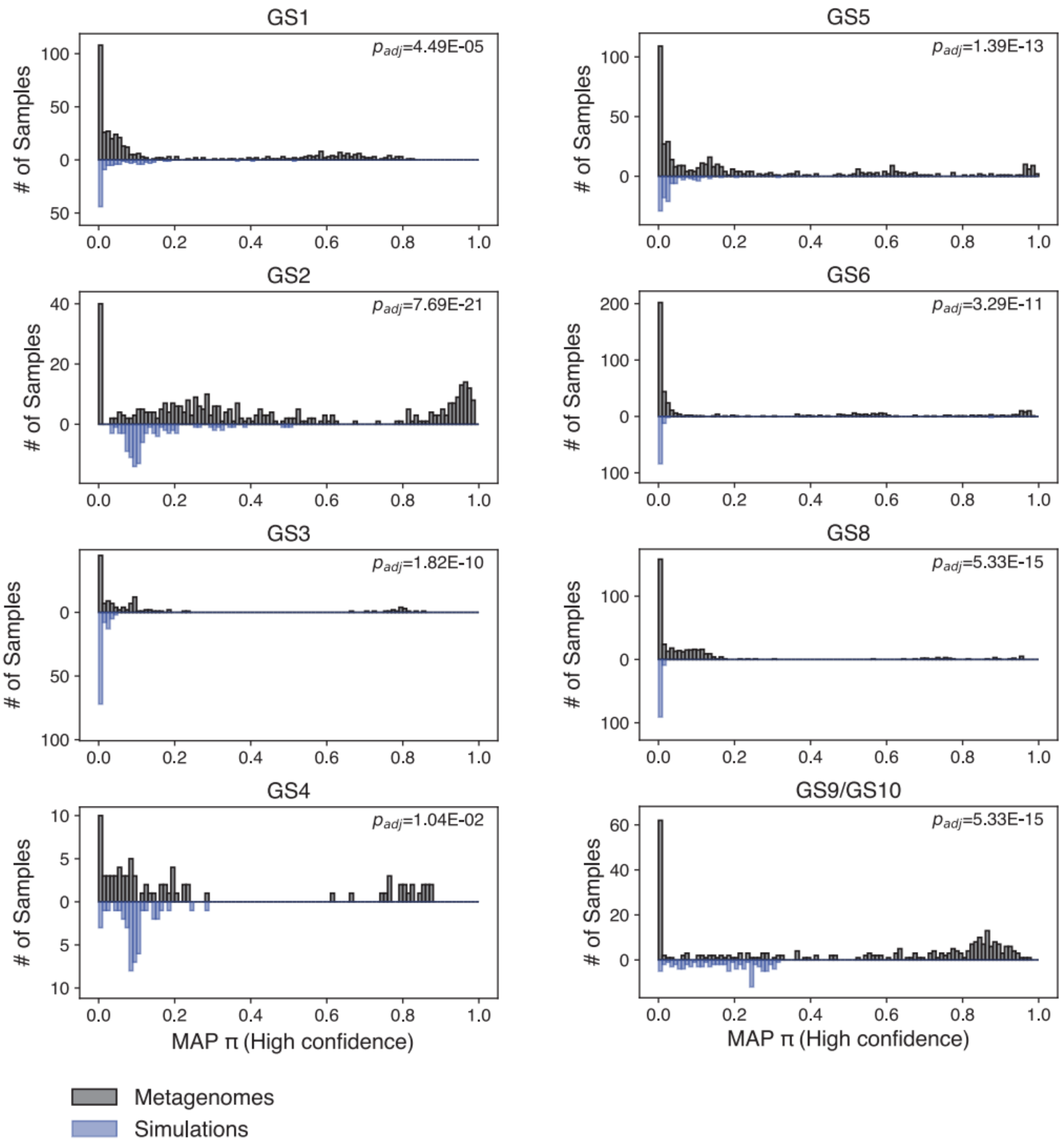

Figure S16: Peaks in high-confidence π values across samples suggest novel *Gardnerella* clades. Histograms showing the distribution of high-confidence maximum a posteriori (MAP) estimates for π, calculated with respect to each defined *Gardnerella* clade. MAP estimates for π are taken from real metagenomes (black) and 100 simulated metagenomes (blue) composed of random combinations of *Gardnerella* genomes. Results from a two-sample K-S test comparing the distribution of π estimates between real and simulated metagenomes are shown next to each graph (Bonferroni-corrected p-values).

bioRxiv preprint doi: https://doi.org/10.1101/2025.02.07.636498; this version posted February 8, 2025. The copyright holder for this preprint (which was not certified by peer review) is the author/funder, who has granted bioRxiv a license to display the preprint in perpetuity. It is made available under aCC-BY 4.0 International license.

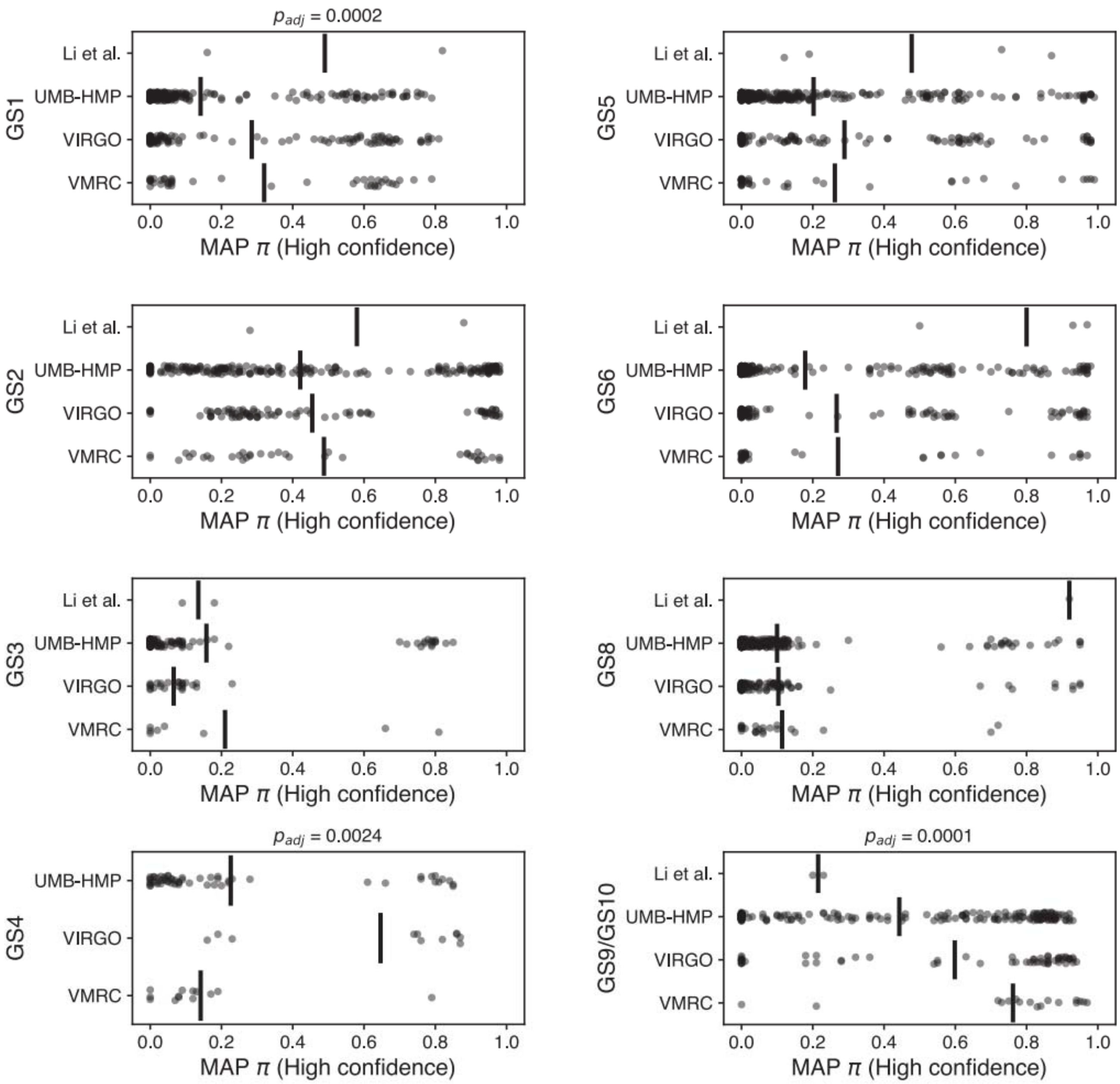

Figure S17: Putative novel *Gardnerella* clades are enriched in specific studies and samples. MAP estimates for π, calculated with respect to each defined *Gardnerella* clade, separated by study. Each dot represents one estimate from one sample; mean lines for studies are shown in bars. Adjusted p-values for clades that have significantly differently distributed π estimates across studies (Kruskal-Wallis test, Bonferroni-corrected).

bioRxiv preprint doi: https://doi.org/10.1101/2025.02.07.636498; this version posted February 8, 2025. The copyright holder for this preprint (which was not certified by peer review) is the author/funder, who has granted bioRxiv a license to display the preprint in perpetuity. It is made available under aCC-BY 4.0 International license.
